# Coherence and multimode correlations from vacuum fluctuations in a microwave superconducting cavity

**DOI:** 10.1038/ncomms12548

**Published:** 2016-08-26

**Authors:** Pasi Lähteenmäki, Gheorghe Sorin Paraoanu, Juha Hassel, Pertti J. Hakonen

**Affiliations:** 1 Department of Applied Physics, Low Temperature Laboratory, Aalto University School of Science, PO Box 15100, FI-00076 Aalto, Finland; 2 VTT Technical Research Centre of Finland, FI-02044 Espoo, Finland

## Abstract

The existence of vacuum fluctuations is one of the most important predictions of modern quantum field theory. In the vacuum state, fluctuations occurring at different frequencies are uncorrelated. However, if a parameter in the Lagrangian of the field is modulated by an external pump, vacuum fluctuations stimulate spontaneous downconversion processes, creating squeezing between modes symmetric with respect to half of the frequency of the pump. Here we show that by double parametric pumping of a superconducting microwave cavity, it is possible to generate another type of correlation, namely coherence between photons in separate frequency modes. The coherence correlations are tunable by the phases of the pumps and are established by a quantum fluctuation that stimulates the simultaneous creation of two photon pairs. Our analysis indicates that the origin of this vacuum-induced coherence is the absence of which-way information in the frequency space.

## Abstract (web summary)

Vacuum fluctuations can produce observable phenomena which can potentially be harnessed, for example using the dynamical Casimir effect. Here, the authors show that, on the basis of the same effect, it is possible to establish distinct two-mode coherence correlations in a pumped microwave cavity owing to absence of which-way information.

## Graphical abstract

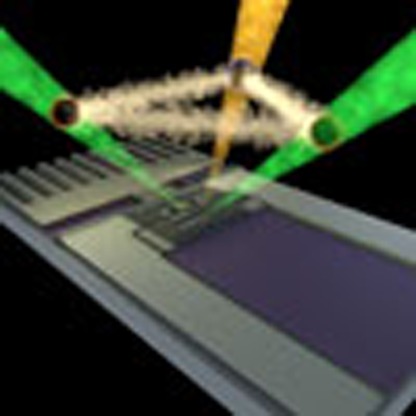

A direct consequence of the Heisenberg uncertainty principle is that quantum fields, even in the vacuum state, are teeming with fluctuations. Modern quantum field theory predicts that these fluctuations are not only a useful mathematical representation but also they can produce observable effects, encompassing vastly different physical scales—from atoms to black holes. Among these are the Purcell effect^1^, the Lamb shift of the atomic states^2^, the Schwinger effect^3^, the Hawking radiation^4^ and the Casimir effect^5^.

Recently, in parallel to the interest in fundamental physics^6^, a novel approach has emerged—the idea of engineering the quantum vacuum to create novel devices and protocols for quantum technologies^7^. Parametrically modulated superconducting circuits have attracted significant interest^8^,^9^,^10^, motivated also by the demand for parametric amplifiers where the added noise is pushed to the quantum limit^11^,^12^,^13^,^14^,^15^. In these systems, it has been demonstrated that vacuum fluctuations present at the input port trigger the creation of real microwave photons^16^,^17^,^18^,^19^,^20^,^21^,^22^. Some of these experiments provide beautiful analogies with the motion of a mirror in free space^20^,^21^,^23^,^24^, also referred to as dynamical Casimir effect. For the development of quantum computing with continuous variables (CV) in microwave circuits^25^, these results demonstrate that a key operation is now available experimentally: two-mode squeezing^26^,^27^ of the modes *a* and *b*, leading to a non-zero correlation 〈*ab*〉≠0. However, there exists another fundamental type of correlation between microwave fields, yielding 〈*a*^†^*b*〉≠0, which is instrumental in CV quantum computing. This is a measure of coherence of the fields in the two modes, and it appears ubiquitously in the modelling of beam-splitter and interference phenomena. From the perspective of quantum vacuum engineering, achieving this correlation is problematic: vacuum fluctuations at different times and frequencies are completely uncorrelated. This is such a precise feature, that vacuum fluctuations can even serve as perfect random number generators^28^. Even when the fluctuations trigger, the downconversion of a higher-energy photon into two photons in modes *a* and *b*, as in the case of a single-pump dynamical Casimir effect, the correlation 〈*a*^†^*b*〉 remains zero.

Here we demonstrate that two-mode coherence correlations can be obtained from the dynamical Casimir effect by employing another fundamental quantum mechanical principle, namely the absence of which-way information^29^,^30^, which is applied here in the frequency space. Specifically, we study the parametric modulation of the quantum vacuum under the action of two pumps. We demonstrate that a nonzero coherent correlation 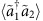 is established between two frequency modes 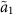 and 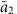, through downconversion processes in the two pumps triggered by the same vacuum fluctuation in a third-frequency mode 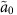. This correlation is non-zero provided that it is not possible to specify from which pump the downconverted photons at mode 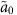 originate from. In addition, due to this coherence effect, a highly populated bright mode and, orthogonal to it, a zero-population dark mode can be defined in the subspace of the two frequency modes. Dark states appear as a result of destructive quantum interference. Typically, in atomic physics, optics and more recently in superconducting microwave systems, dark states appear in Rabi-driven multilevel quantum systems^31^,^32^,^33^,^34^,^35^,^36^, or, in the case of resonators, through sideband driving^37^,^38^, both of which require Hamiltonians of the type *a*^†^*b*+*h*.*c*. to simply transfer population from one level to another. In contrast, here our Hamiltonian is of parametric type *a*^†^*a*^†^+*h*.*c*., which creates populations from the vacuum instead of transferring quanta between modes. We also show that this parametric coherence effect is a phase-sensitive phenomenon and, therefore, it can be controlled by the relative phase of the applied pumps. Our result opens a way for realizing highly complex multi-mode entangled states displaying both coherence and squeezing correlations with CV. In terms of the modes 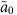, 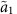 and 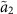, the state we created is a CV tripartite state^39^,^40^ of bisymmetric type^41^,^42^, which at low powers, in the subspace with at most two photons, becomes a W-type state^43^. Tripartite bisymmetric states are a resource for protocols such as one-to-two telecloning^44^,^45^, while W states can be used for fundamental tests of quantum mechanics^46^,^47^. Our approach can be generalized in a straightforward way to multiple modes and pumps, opening the way to the implementation of algorithms such as bosonic sampling^48^ and the realization of CV cluster states for one-way quantum computing^49^,^50^ in the microwave regime.

## Results

### Theoretical predictions

**Figure 1:**
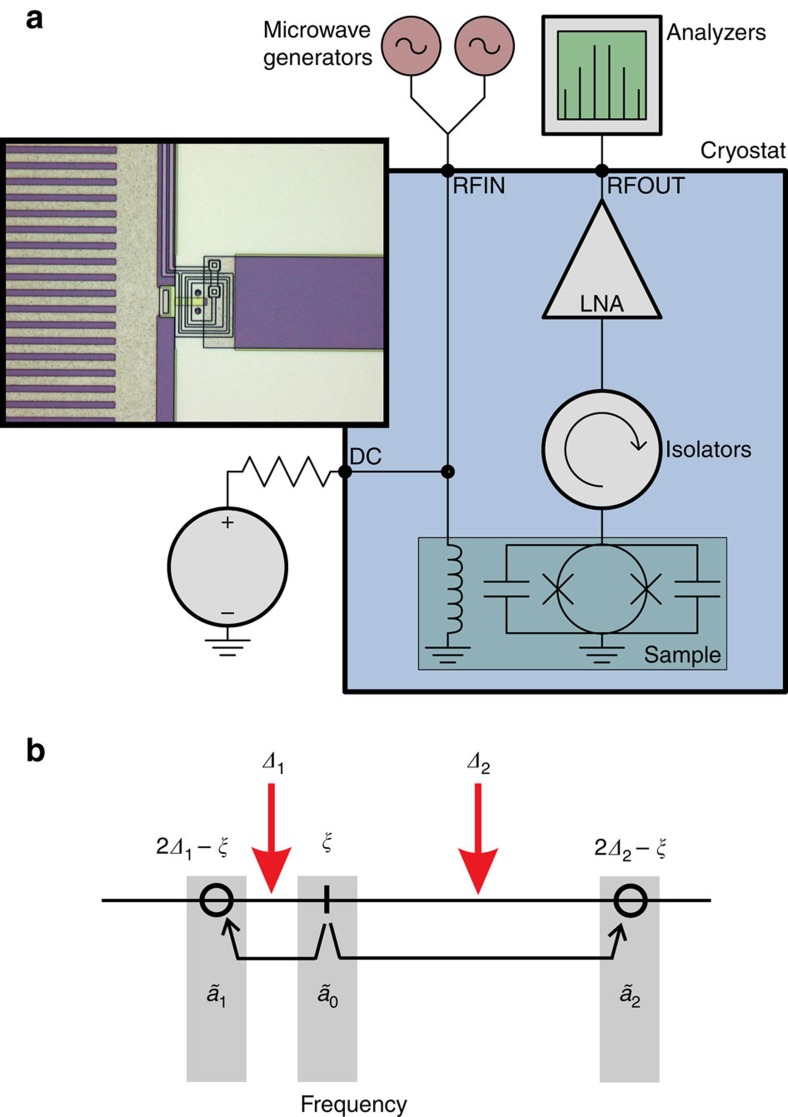
Overview of the measurement setup and pumping. (**a**) Simplified schematic representation of the measurement setup (see Supplementary Figs 3 and 4 from Supplementary Note 7 for more detailed schematics). The investigated resonator was made using a capacitively shunted superconducting quantum interference device (SQUID), fabricated using a NbAlO_*x*_Nb trilayer process at VTT^57^. The device forms a flux tunable resonator which acts as a tunable boundary condition for the reflected waves in the attached 50 Ω transmission line^21^. The resonance is tuned down from the maximum value (nominally 10 GHz) to 5 GHz using dc-flux biasing. The designed critical current for the junctions of the SQUID was *I*_*C*_=33 μA, and the total parallel capacitance was *C*=40 pF. We estimate the effective temperature to remain below 35 mK at the highest pumping powers used in these experiments while the cryostat base temperature is held at 15 mK. This corresponds to thermal occupation number 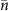
=10^−3^. Modulation of the flux through the SQUID is realized through a lithographically fabricated rectangular spiral coil underneath the junction layer. (**b**) Illustration of the first reflections of a frequency *ξ*=*ω*−*ω*_res_ with respect to half the pump frequencies. The short-hand notation of modes 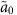, 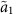, and 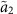 signifies the cavity output modes 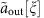, 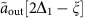, and 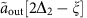, respectively.

Our system consists of a superconducting resonator (see Fig. 1) which can be described by the effective Hamiltonian^21^


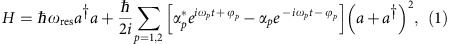


where *α*_*p*_ is the strength of pump *p* coupled into the resonator, in units of frequency, and 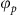 specifies the pump phase. Denoting the decay rate of the resonator by *κ* and setting *ξ*=*ω*−*ω*_res_, the output field 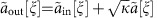 is obtained within the input–output formalism by solving iteratively the Heisenberg–Langevin equations (HLEs) in a rotating frame at the resonator frequency defined by 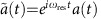. We obtain a nested structure


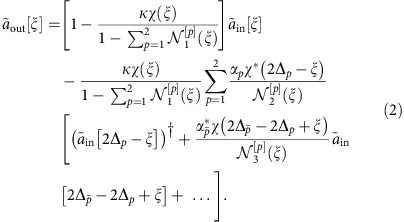


Here *χ*(*ξ*)=(*κ*/2−*iξ*)^−1^ is the electrical susceptibility of the resonator, 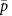
∈{1, 2}\{*p*}, 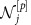 denote normalization factors (see the Supplementary Notes 1–3), and Δ_*p*_=*ω*_*p*_/2−*ω*_res_.

For simplification, we truncate equation (2) to the first-order reflections of ξ with respect to the pumps, and assume |Δ_*p*_|≫|*ξ*|. Higher-order reflection (Supplementary Fig. 1) amplitudes are suppressed by a factor proportional to the cavity susceptibility, however they can be observed experimentally at high enough pumping power (Supplementary Fig. 6). In this first-order approximation, we have 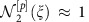, and we use a pump-power parameterization by a squeezing parameter *λ* and power asymmetry angle *θ*, 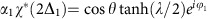, and 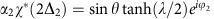. This allows us to calculate the correlations in the output field of the cavity 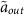, with the vacuum as the input state. We find that each pump produces squeezing correlations 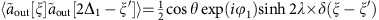, 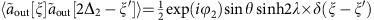 as well as noise at 2Δ_1_−*ξ* and 2Δ_2_−*ξ*, according to the dynamical Casimir effect power noise formula^21^ for each pump separately, 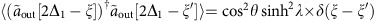, 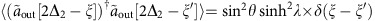, while at ξ the noise is produced by the action of both pumps, 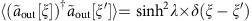. Surprisingly, the simultaneous action of the two pumps produces the correlation 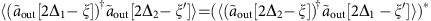 between the frequencies 2Δ_1_−*ξ* and 2Δ_2_−*ξ*,


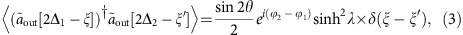


which can be regarded as a coherence between the extremal modes 2Δ_1_−*ξ* and 2Δ_2_−*ξ*, mediated by the middle vacuum fluctuation. From the expression of the correlations above we can construct the covariance matrix, which has a bisymmetric structure^41^,^42^. Note that, since the Hamiltonian is quadratic in the field operators, the state is Gaussian. Therefore, the first-order correlations presented here provide a complete characterization of the output field.

On the basis of the structure of equation (2) we can introduce ‘bright' and ‘dark' modes,


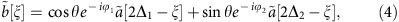



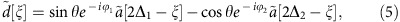


and similar relations for the output and input modes. These two modes are orthogonal to each other, spanning the Hilbert space of the extremal modes 2Δ_1_−*ξ* and 2Δ_2_−*ξ*. Experimentally, the asymmetry angle *θ* in the bright and dark modes is determined from measurements of the noise power generated by each pump through the dynamical Casimir effect. The definition of the modes 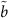 and 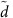 resembles a beam-splitter/merging operation in frequency (rather than in space, as in usual interferometers), where a mode is separated into two branches with a distance 2(Δ_1_−Δ_2_) between them. Indeed, when the modes 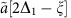 and 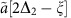 are rotated by some angles 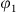 and 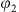 and combined as in equations (4) and (5), this vacuum-induced coherence manifests as an interference effect, producing the extinction of power in the dark mode and the maximization of power in the bright mode. Specifically, for the correlations involving the dark and bright modes we obtain


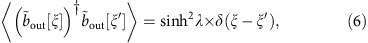



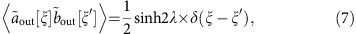



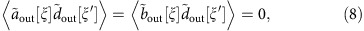



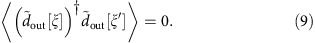


These correlations reflect the structure of the output state of the resonator |vac〉_out_, which fulfils 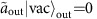. For the truncated form of equation (2), the output state is a two-mode squeezed state in terms of the operators 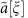 and 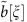, 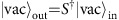, where 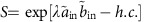. In the subspace containing at most two excitations, the tripartite state is in the W class, see Supplementary Note 5.

The disappearance of power in the dark mode is a particular manifestation of coherence which has been employed for many applications, such as quantum memory^35^ and stimulated Raman adiabatic passage (STIRAP) in superconducting qubit systems^36^. Note that in the usual type of dark state, the destructive interference is produced at the same frequency, for example, on a quantum state of a qubit or of a mode of a field. Here, the two modes at 2Δ_1_−*ξ* and 2Δ_2_−*ξ* are separated in frequency and do not overlap^51^. The destructive interference effect is created by the vacuum fluctuations at ξ which trigger two correlated two-photon parametric downconversion processes in the pumps.

As for standard quantum interference, the lack of path information^52^,^53^ is critical for the interference between extremal frequencies. Here, instead of which-path information in space, we deal with absence of which-colour information^29^,^30^: for a real photon at frequency ξ, there is no way of knowing from which of the two spontaneous parametric downconversion processes it came from (see Supplementary Note 4).

### Experimental results for correlations

**Figure 2:**
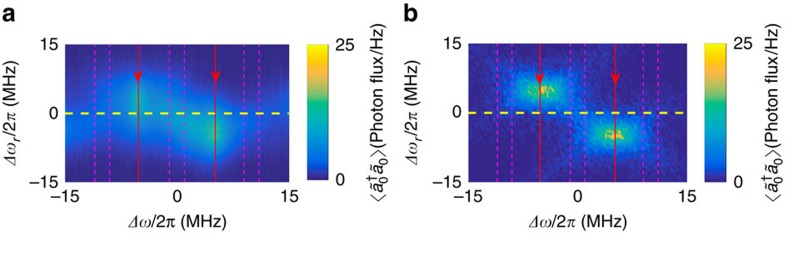
Noise power spectra. (**a**) Measured noise power spectra corresponding to the correlator 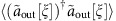 for fixed half-pump frequencies *ω*_1_/2=(2*π*) × 4.995 GHz, *ω*_2_/2=(2*π*) × 5.005 GHz at different values of the resonance frequency Δ*ω*_r_/2*π* and measurement frequency Δ*ω*/2*π* relative to (*ω*_1_/2+*ω*_2_/2)/2=(2*π*) × 5 GHz for a pump power corresponding to 20 photon flux/Hz emerging from the cavity at the centre frequency of 5 GHz. The colour bar is scaled in dB relative to vacuum. The dashed magenta lines indicate the measurement bands within which the correlation data were collected. The red lines indicate the locations of the half of the pump frequencies *ω*_1_ and *ω*_2_. The dashed yellow line indicates the symmetry point which was used for measuring the data depicted in Fig. 3. The external and internal cavity decay rates for this sample were *κ*_*E*_=2*κ*_*I*_≈36 MHz. (**b**) Noise power as predicted by equation (2) including only first-order reflections with *κ*=*κ*_*E*_+*κ*_*I*_ taken from the measurement (Supplementary Fig. 2).

To test the above predictions, we pumped our sample using two phase-locked microwave generators at frequencies 9.99 and 10.01 GHz. The output field 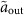 from the resonator is amplified, yielding a signal that is further downconverted and digitized. The Fourier-transformed amplified field is measured in a bandwidth around the three frequencies of interest. The resulting fields are denoted by 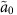, 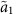, 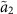 (see Methods), corresponding to the output fields 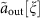, 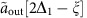 and 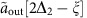, respectively. Upon substraction of the added noise of the amplifiers, the correlations between the amplified fields provide a direct measurement of the corresponding correlations of the resonator output field^19^,^54^. For additional information see Supplementary Notes 7–8. The measured noise power data can be reproduced by simulations using the theoretical results presented earlier. The data are presented in (Fig. 2a), while the corresponding numerical result from equation (2) is presented in Fig. 2b). The measured correlations, as well as the analytical and the simulation results are normalized with respect to the vacuum state values, corresponding to *λ*=0. Thus, the correlations at finite pumping *λ*≠0 are expressed in dB (with vacuum as reference). The noise power levels can also be expressed in absolute units (photon flux/Hz).

For symmetry reasons, we chose to analyse correlations when the microwave resonance lies at half of the average of the two pump frequencies, that is Δ_1_≈−Δ_2_, in which case the tripartite correlations are strongest.

**Figure 3:**
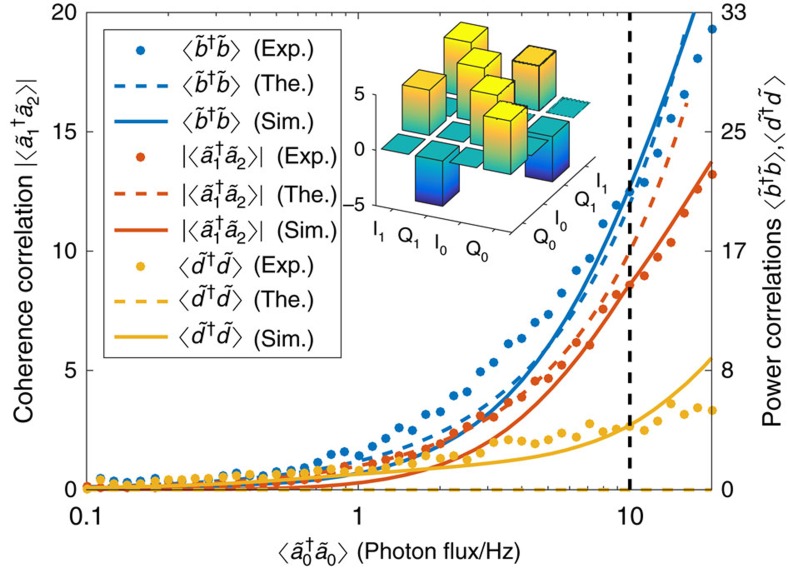
Experimental correlations and theoretical predictions. We present the measured correlators (specified in the inset label) versus photon flux/Hz at the output of the cavity measured at the centre frequency of 5 GHz; see text for the relation between the photon flux and the squeezing parameter. The symbols refer to measured data, while the curves are predictions from theory (dashed) and simulations (solid). Our simulation includes the higher order reflections and it reproduces also the residual population in the dark mode. The 3D histogram represents the measured covariance matrix corresponding to the fields 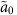 and 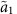, where the mode quadratures defined as 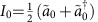, 
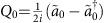
, 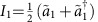 and 
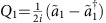
, yield two-mode squeezing correlations 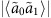 across the first pump. For additional correlations see Supplementary Fig. 5.

Figure 3 displays the most relevant correlators determined as a function of the number of photons in the resonator. All these correlators can be calculated from the measured field quadratures of the output field. The simulation is based on the Langevin equation corresponding to the Hamiltonian equation (1) (see also equation (6) in Supplementary Note 1), with white noise as input, and the theoretical curves are obtained from equations (3), (6) and (9). We find that the behaviour of these correlators agrees well with the theory—squeezing correlations exist between neighbouring frequencies and vacuum-induced coherence correlations between the extremal ones. Indeed, the correlators grow nearly exponentially with the squeezing parameter, as predicted by theory. Furthermore, the ratio between various correlators is close to the expectations obtained from equation (2), with only the first-order pump reflections included.

From Fig. 3 we note that the coherence is preserved also in the limit of small number of photons. This shows that our result is fundamentally different from tripartite slit-interferometer schemes^55^, where the coherence between two modes is obtained only in the limit of a large number of photons in the pump mode.

**Figure 4:**
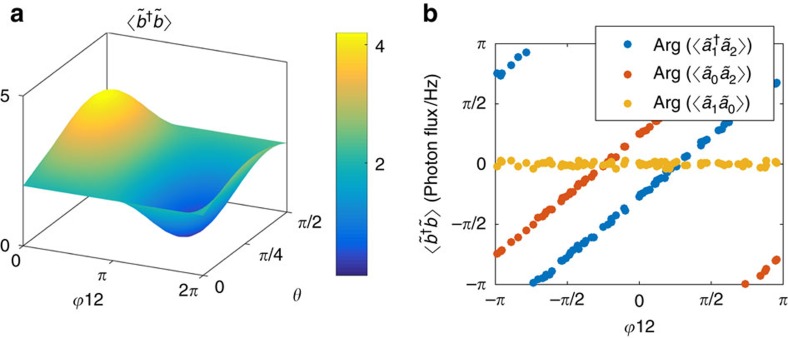
Phase sensitivity of correlations. (**a**) Bright-/dark-mode amplitude versus phase difference between the pumps 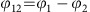 and pump amplitude asymmetry parameter *θ* (symmetric pumps have *θ*=*π*/4). The maximum value corresponds to the bright-mode amplitude while the minimum refers to the dark mode. (**b**) Measured argument of the complex quadrature correlations versus 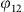, where 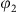 is varied and *θ* ≈ *π*/4.

The phase-dependent interplay between the bright and dark modes is presented in (Fig. 4a). By adjusting the phase difference 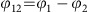 of the pumps, the measured correlator power will shift from 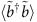 into 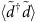 as seen in Fig. 4a. The division of power between the dark and bright modes depends also on the asymmetry of the two pump amplitudes, that is, the parameter *θ*. Thus, we find *θ* by measuring the noise power generated by each pump separately. Figure 4b illustrates the change in the argument of the correlator when the phase of one pump is varied relative to the other one. We note that the possibility of applying phase shifts on the modes by the rotation of the pump phases is a key operation in CV quantum computing^56^.

### Correlations in time-domain

**Figure 5:**
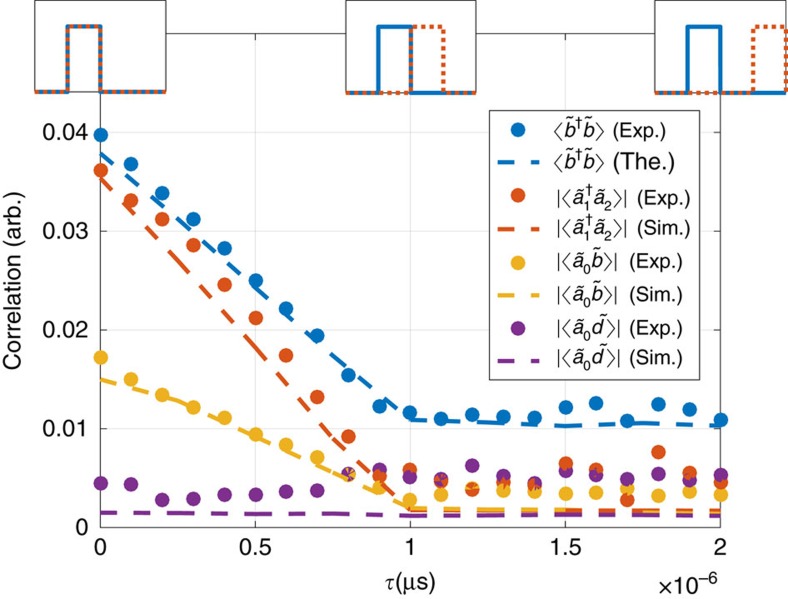
Pulsed-pump measurements. Decrease of vacuum-induced coherence 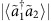 when the overlap time *τ* of the parametric pump pulses is varied. The coherence decreases linearly (see Supplementary Note 6) as the pumps become non-overlapping. Complete vanishing of the correlation between the extremal frequencies with zero overlap of the pulses implies that the photons are created simultaneously. For this experiment the sample was replaced with a new higher *Q* chip with 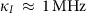, *κ*_*E*_≈2 MHz. The pulse width for both pump tones was 1 μs. Three other correlators specified in the inset labelling are also depicted. Dashed lines are from time domain simulations based on equation (6) from the Supplementary Note 1 and the dots are experimental data. The difference in the values of 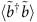 and 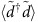 when the pulses do not overlap is due to the asymmetric power generation by the dynamical Casimir effect in each of the pumps separately.

The creation of vacuum-induced coherence was investigated in pulsed pump tone experiments, in which the overlap of the pump signals was varied as illustrated in (Fig. 5. The results show that, in order to obtain a non-zero coherence correlation, the downconversion processes have to occur simultaneously. We observe that the coherence 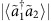 is reduced proportionally to the decrease of pulse overlap, while the squeezing correlations 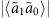 and 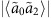 remain. The linear dependence of the coherence correlation on the overlap can be obtained through a fully analytical relation (see Supplementary Note 6). This demonstrates that the generation of the coherence requires overlapping pump signals and simultaneous creation of photons, during which the which-colour information is not available. When the pump pulses are separated in time, the information about which pump has generated the photons becomes accessible in principle, and the coherence is suppressed.

## Discussion

Our work on tripartite microwave correlations can be extended towards multipartite entangled states^49^, which would yield a platform for universal quantum computation using CV, as recently proposed in ref. 25. For example, the creation of cluster states in microwave cavities would be an important step towards realizing a superconducting one-way computer. Such multipartite entangled states require pulsed microwave pumping for entangling different frequencies, a scheme that has been shown to be a fully functioning concept in our work.

## Methods

### Numerical evaluation of correlators

The numerically simulated results presented in Figs 2 and 3 were obtained using Gaussian (white) noise to represent the effect of the vacuum as input. In addition, higher-order reflections were included in Fig. 3 by solving the HLE in the rotating-wave approximation 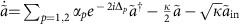. The presumption of white noise is accurate for narrow bandwidths where the spectrum of quantum noise is approximately uniform. In this way, higher-order reflections are included automatically in the results.

As seen from Fig. 3, the simple tripartite analytical solution of equation (2), leading to correlators listed in equations (5)–(9), 35, 36, 37, 38, agrees quite well with the results, except for the dark-mode correlator 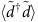. The disagreement can be traced to the assumption 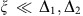, but the numerical simulations are not limited by this approximation.

### Homodyne detection of correlations

The output field of the resonator propagates through circulators and is amplified by a cryogenic low-noise high-electron-mobility transistor (HEMT) amplifier and by room-temperature microwave amplifiers. The quadratures are measured by standard homodyne methods, that is, captured using an Anritsu MS2830A signal analyser. Approximately 20 GB of data per sweep were collected and transferred to PC for later analysis. The single bins of the Fourier transformed data represent a bandwidth of 50 Hz and, therefore, spectral leakage due to used rectangular windowing is insignificant.

From the quadratures, we can construct the complex field amplitudes, calculate their Fourier components around the central and extremal frequencies, in a bandwidth *BW*. We can write


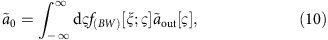



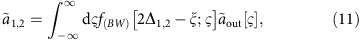


where *f*_(*BW*)_[*ξ*;*ς*] is a digital filtering function of width *BW* centred at the frequency ξ. Choosing 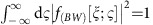 ensures that 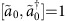, 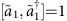, 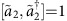. A simple choice is 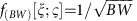, if *ς* is in the interval (*ξ*−*BW*/2, *ξ*+*BW*/2), and 0 otherwise.

Because the added noise of the measurement chain is contained in 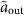 and it is uncorrelated, its contribution will vanish when calculating the correlations of 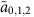 and in the case of autocorrelation, it can be subtracted as a known constant. We can obtain then directly from equations (10) and (11) all the measured correlations, for example 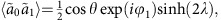 etc.

Also, given the definitions above, the correlations in the field at the output of the cavity are adimensional; in particular, 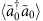, 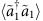 and 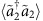 represent the flux of photons per bandwidth in the modes 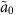, 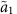 and 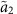, respectively. This power is estimated from the increase of observed power due to pumping relative to the amplifier-noise temperature.

### Pulsed parametric pumping

In pulsed measurements, the pumping tones (corresponding to 50 photon flux/Hz in continuous operation emerging from the cavity at the centre frequency of 5 GHz) were modulated using a pair of mixers (Marki M10220LA), driven by digital delay generators from Stanford Research System (DG535). The system provides an ON/OFF ratio of 1:100 and a rise time of 10 ns. The repetition rate was set to 250 kHz, which resulted in signals of 30% when compared with continuous measurements. With a pulse width of 1 μs, the repetition rate corresponds to a duty cycle of 1:3. The same method of simulating the HLEs in time domain was employed to calculate numerically the response for pulsed pump tones.

### Data availability

The data that support the findings of this study are available from the corresponding author upon request.

## Additional information

**How to cite this article:** Lähteenmäki, P. *et al*. Coherence and multimode correlations from vacuum fluctuations in a microwave superconducting cavity. *Nat. Commun.* 7:12548 doi: 10.1038/ncomms12548 (2016).

## Supplementary Material

Supplementary Information Supplementary Figures 1-6, Supplementary Notes 1-8 and Supplementary References.
