# Supplementary material for: Coherence and multimode correlations from vacuum fluctuations in a microwave superconducting cavity
Source: Nat Commun. 2016 Aug 26;7:12548. doi: 10.1038/ncomms12548 (PMC5007450; doi:10.1038/ncomms12548)
Supplement: Supplementary Information — Supplementary Figures 1-6, Supplementary Notes 1-8 and Supplementary References. [file ncomms12548-s1.pdf]

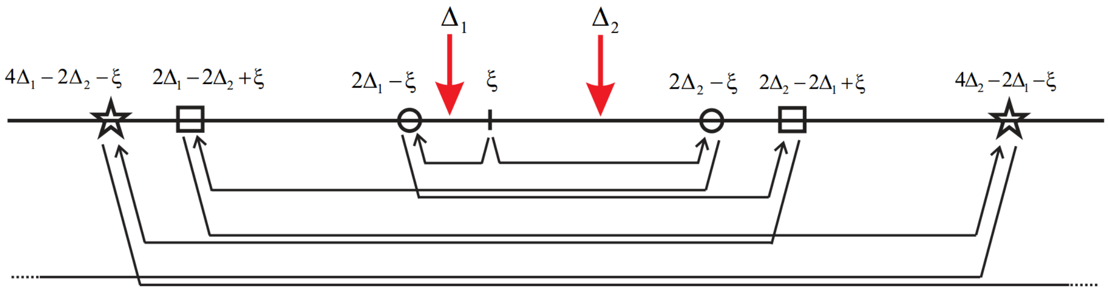

Supplementary Figure 1: **Schematic of the reflections up to the third order with respect to half of the pump frequencies.** The two pumps are indicated with red arrows. A frequency ξ is reflected to $2\Delta_1 - \xi$ with respect to the first pump, then to $2\Delta_2 - 2\Delta_1 - \xi$ with respect to the second pump, *etc.*

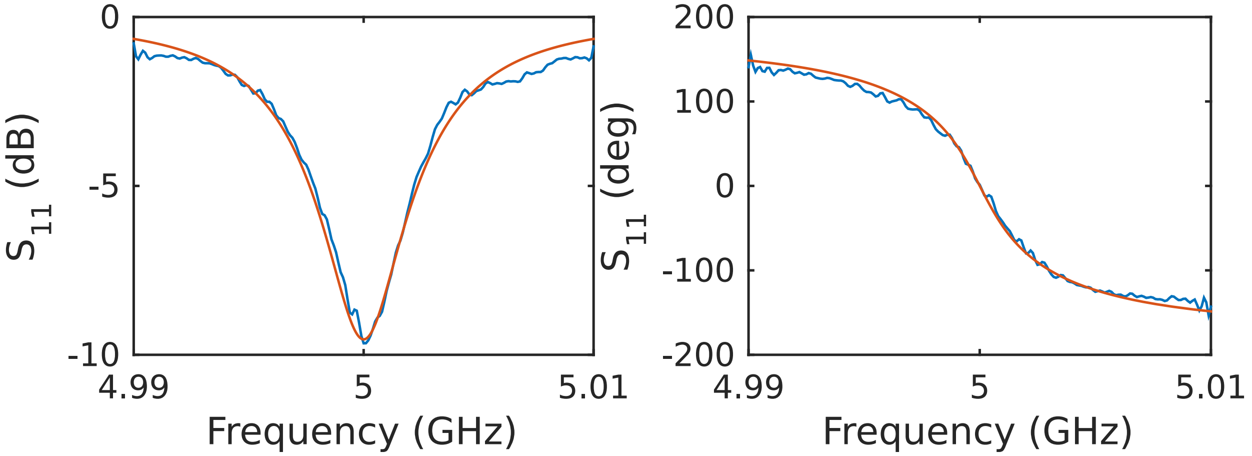

Supplementary Figure 2: **Reflection measurement.** Extraction of the internal and external cavity decay rates κ_I and κ_E from reflection measurements, see Eqs. 15-17 for definitions. For these measurements the pumps are turned off and a classical input field supplied. The fitting is done using the standard input-output theory for dissipative cavities, see [10]. The measurement yields a fit $\kappa_{\mathrm{E}} \approx 2\kappa_{\mathrm{I}} \approx 36$ MHz.

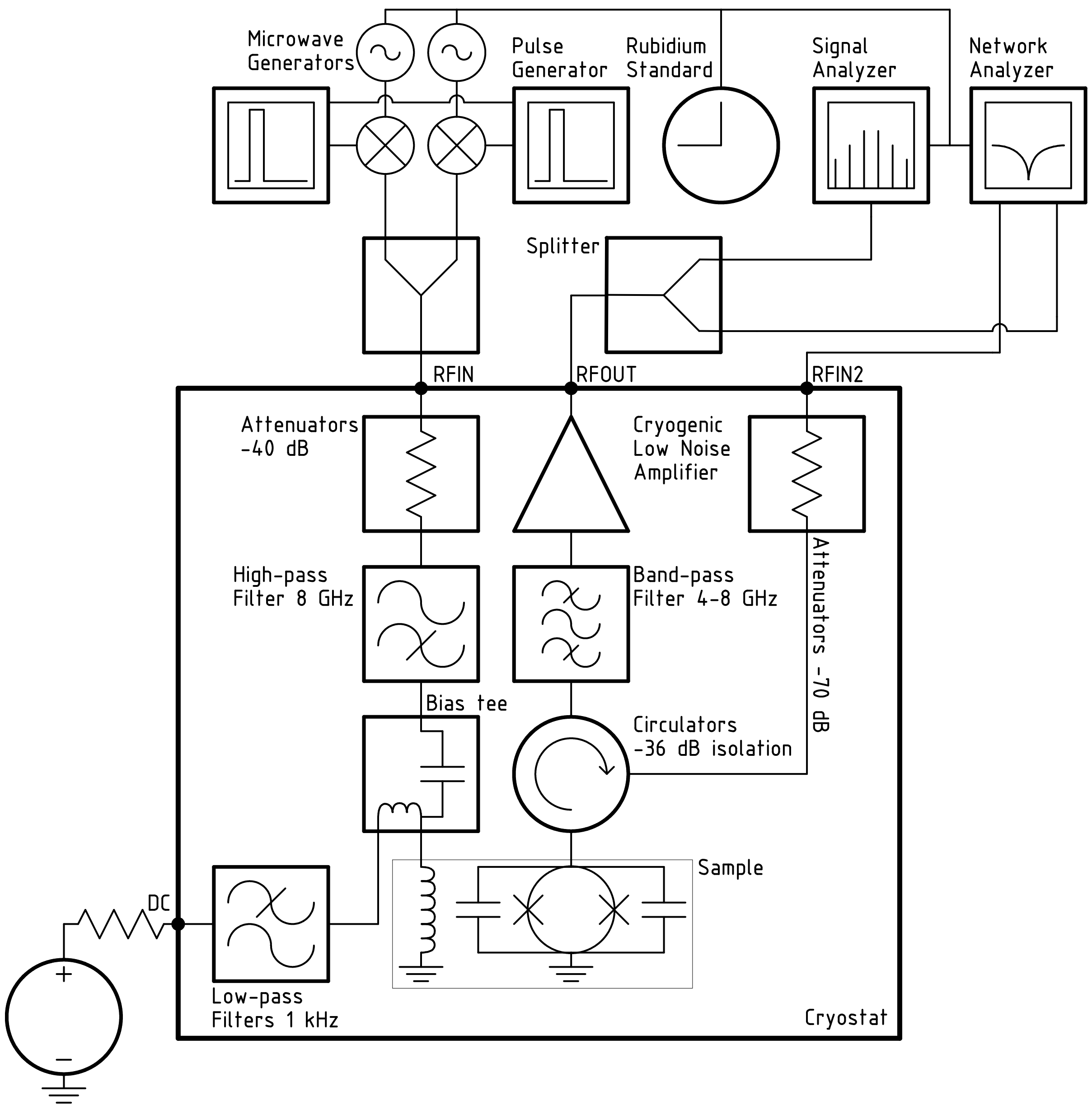

Supplementary Figure 3: **Schematic of the measurement.**

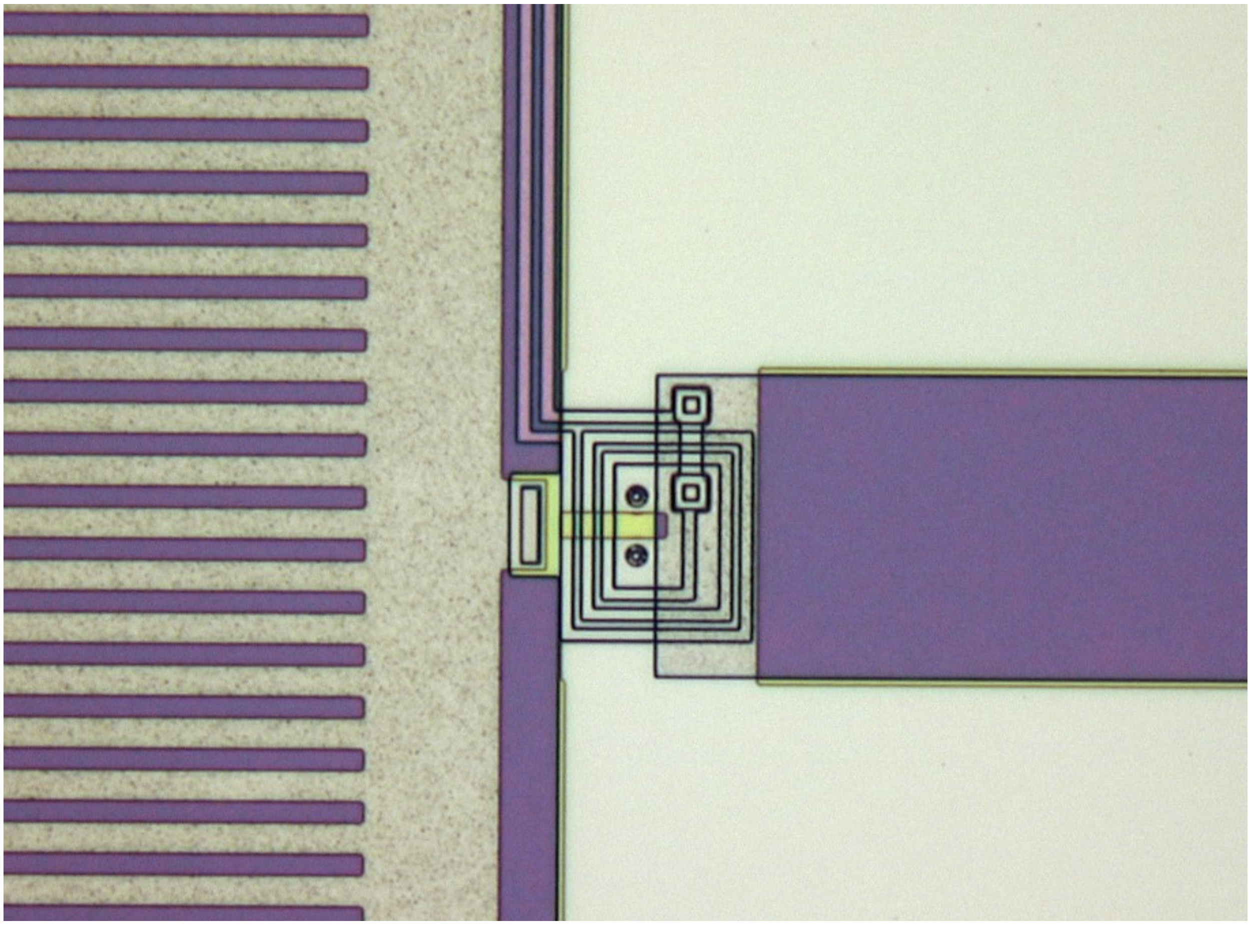

Supplementary Figure 4: **A micrograph of the sample.** The comb-like structure on the left is a bonding pad and the pump coil along with its vias is seen as the most prominent structure in the middle. The large solid pads up and down are symmetric parallel plate capacitors. The two Josephson junctions are seen as two round dark circles.

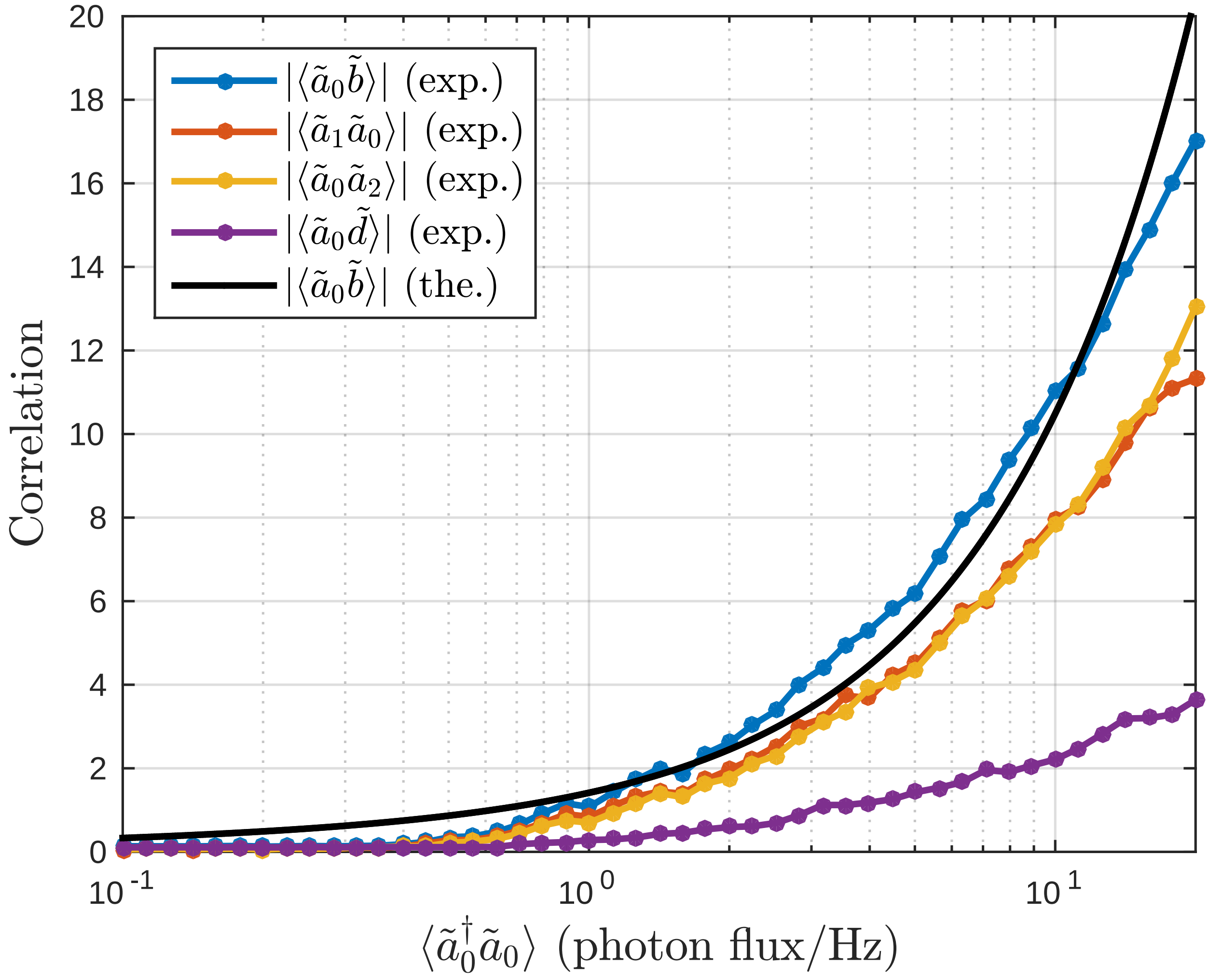

Supplementary Figure 5: **Experimentally measured squeezing correlations.** We present here the correlators involving coupling between the cavity mode to the bright and dark modes vs. photon flux/Hz emerging from the cavity at the center frequency of 5 GHz. Squeezing correlations between $\tilde{a}_{\rm out}[2\Delta_1 - \xi]$ and $\tilde{a}_{\rm out}[\xi]$, as well as between $\tilde{a}_{\rm out}[\xi]$ and $\tilde{a}_{\rm out}[2\Delta_2 - \xi]$ are also indicated.

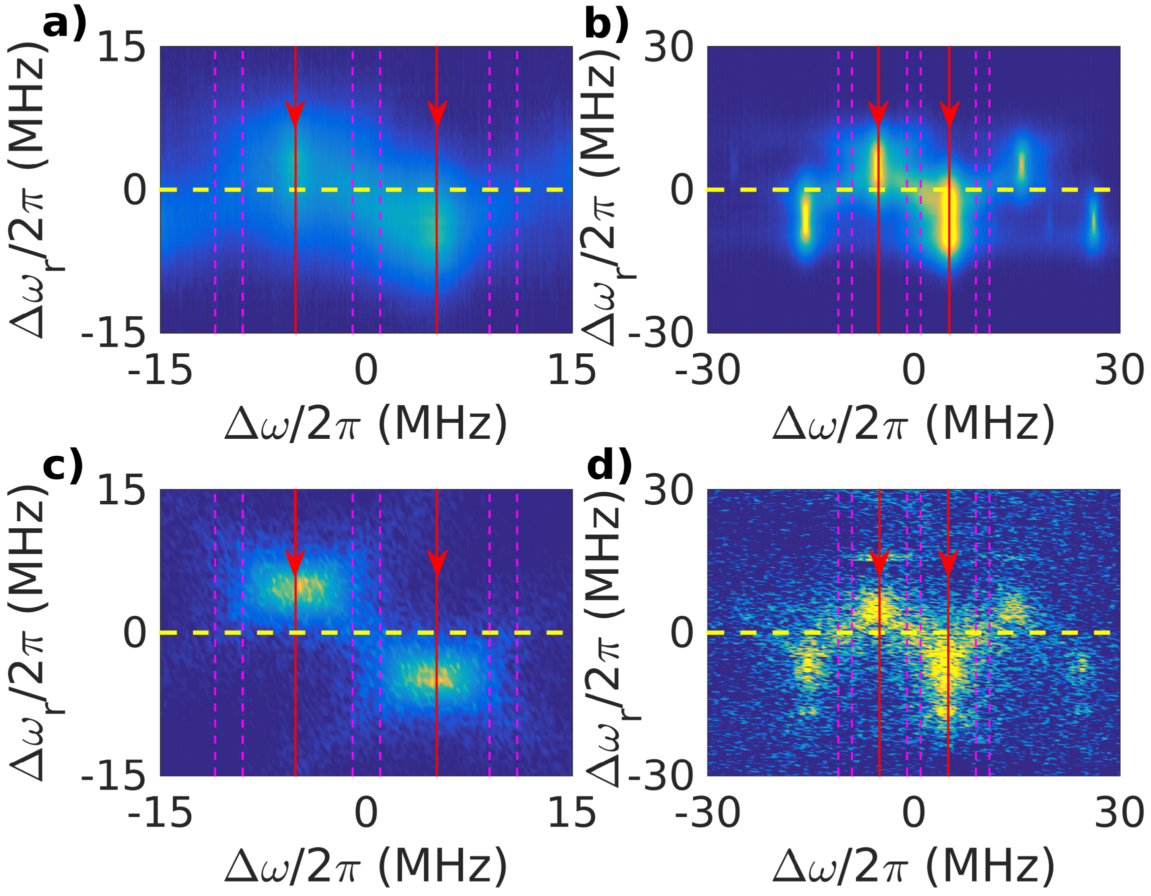

Supplementary Figure 6: **Observation of higher-order reflections.** a) and c) show the low-power pump spectra presented in the main paper Figure 2. with all the same markings. b) Higher pump power corresponding to 100 photon flux/Hz emerging from the cavity at the center frequency of 5 GHz. d) Numerical simulation of the expected noise power corresponding to b) including higher order reflections (see Methods).

Supplementary Note 1: Derivation of the Heisenberg-Langevin equations of motion

In our analysis we define the Fourier transform as

$$a[\omega] = \frac{1}{\sqrt{2\pi}} \int_{-\infty}^{\infty} dt e^{i\omega t} a(t), \tag{1}$$

and use the conventions of Ref. [1] for the Fourier transform of the conjugate operator

$$a^\dagger[\omega] = (a[-\omega])^\dagger = \frac{1}{\sqrt{2\pi}} \int_{-\infty}^{\infty} dt e^{i\omega t} a^\dagger(t). \tag{2}$$

The commutation relations $[a(t), a^\dagger(t')] = \delta(t - t')$ read in the Fourier space $[a[\omega], (a[\omega'])^\dagger] = \delta(\omega - \omega')$. The effective Hamiltonian of the circuit is

$$H = \hbar\omega_{\rm res} a^\dagger a + \frac{\hbar}{2i} \sum_{p=1,2} \left[\alpha_p^* e^{i\omega_p t} - \alpha_p e^{-i\omega_p t}\right] (a + a^\dagger)^2, \tag{3}$$

where ω_p with $p = 1, 2$ are the two pump frequencies. Equivalently, one can replace the two pumps by a single pump operating at the average frequency $(\omega_1 + \omega_2)/2$ but with the amplitude $\alpha_1 \exp[-i(\omega_1 - \omega_2)t/2] + \alpha_2 \exp[i(\omega_1 - \omega_2)t/2]$, that is, the amplitude would be modulated at the half-difference frequency $(\omega_1 - \omega_2)/2$. Let us introduce the corresponding detunings with respect to the frequency of the resonator,

$$\Delta_p = \frac{\omega_p}{2} - \omega_{\rm res}. \tag{4}$$

The dynamics of the system can be solved by writing the Heisenberg-Langevin equations,

$$\dot{a} = -i\omega_{\rm res} a + \sum_{p=1,2} \left[-\alpha_p^* e^{i\omega_p t} + \alpha_p e^{-i\omega_p t}\right] (a + a^\dagger) - \frac{\kappa}{2} a - \sqrt{\kappa} a_{\rm in}, \tag{5}$$

where κ is the external decay rate of the cavity and $a_{\rm in}$ is the input mode. Next, we write Eq. 5 in a frame rotating at the frequency of the resonator with $\tilde{a} = e^{i\omega_{\rm res} t} a$, $\tilde{a}_{\rm in} = e^{i\omega_{\rm res} t} a_{\rm in}$. In the rotating-wave approximation (RWA), by neglecting the terms rotating with high frequencies $\pm\omega_p, \pm(\omega_p + 2\omega_{\rm res})$ we obtain the equation for the slow component of the field

$$\dot{\tilde{a}} = \sum_{p=1,2} \alpha_p e^{-2i\Delta_p} \tilde{a}^\dagger - \frac{\kappa}{2} \tilde{a} - \sqrt{\kappa} \tilde{a}_{\rm in}. \tag{6}$$

As in the main paper, we define the Fourier transform with respect to the cavity resonance, with the variable ξ for $\tilde{a}$ and ω for a, such that $\xi = \omega - \omega_{\rm res}$, resulting in $\tilde{a}[\xi] = \int_{-\infty}^{\infty} e^{i\xi t}\tilde{a}(t) = a[\omega]$. We obtain

$$\left(\frac{\kappa}{2} - i\xi\right)\tilde{a}[\xi] - \sum_{p=1,2}\alpha_p \tilde{a}^\dagger[\xi - 2\Delta_p] = -\sqrt{\kappa}\tilde{a}_{\rm in}[\xi]. \tag{7}$$

We note that the procedure above is equivalent to approximating the Heisenberg-Langevin equation Eq. (5) as

$$\dot{a} = -i\omega_{\rm res}a + \sum_{p=1,2}\alpha_p e^{i\omega_p t}a^\dagger - \frac{\kappa}{2}a - \sqrt{\kappa}a_{\rm in}. \tag{8}$$

Supplementary Note 2: Continued fraction solution of the Heisenberg-Langevin equations of motion

Eq. (7) can be solved iteratively, by starting at a point ξ, obtaining the first-order reflections $2\Delta_1 - \xi$ and $2\Delta_2 - \xi$ with respect to the pumps, then writing again Eq. (7) at $2\Delta_1 - \xi$ and $2\Delta_2 - \xi$ with second-order reflections $2\Delta_2 - 2\Delta_1 + \xi$ (with respect to the second pump) and $2\Delta_1 - 2\Delta_2 + \xi$ (with respect to the first pump), and so on. To simplify the results, let us introduce the cavity electrical susceptibility $\chi(\xi)$, defined with respect to the cavity resonance $\omega_{\rm res}$,

$$\chi(\xi) = \frac{1}{\kappa/2 - i\xi}, \tag{9}$$

with $\xi = \omega - \omega_{\rm res}$. Let us denote by $\bar{p}$ the relative complement of p in the set $\{1,2\}$, $\bar{p} \in \{1,2\} \setminus \{p\}$. A very useful notation is the normalization factor $\mathcal{N}_k^{[p]}(\xi)$ where k is an index counting the number of reflections, and the superscript $[p]$ is the pump with respect to which the first reflection is performed. We note that these normalization factors appear naturally whenever a multimode system is studied in the input-output theory, see *e.g.* Ref. [11]. Then the normalization factor is defined iteratively

$$\mathcal{N}_1^{[p]}(\xi) = 1 - \frac{|\alpha_p|^2\chi(\xi)\chi^*(2\Delta_p - \xi)}{\mathcal{N}_2^{[p]}(\xi)}, \tag{10}$$

and

$$\mathcal{N}_2^{[p]}(\xi) = 1 - \frac{|\alpha_{\bar{p}}|^2\chi^*(2\Delta_p - \xi)\chi^*(2\Delta_{\bar{p}} - 2\Delta_p + \xi)}{\mathcal{N}_3^{[p]}(\xi)}. \tag{11}$$

The next iterations are obtained as $\mathcal{N}^{[p]}_{2k+1}(\xi) = \mathcal{N}^{[p]}_{1}(2^k\Delta_{\bar{p}} - 2^k\Delta_p + \xi)$, and $\mathcal{N}^{[p]}_{2k}(\xi) = \mathcal{N}^{[p]}_{2}(2^k\Delta_{\bar{p}} - 2^k\Delta_p + \xi)$. These normalization factors are a generalization of similar factors introduced in the discussion of the dynamical Casimir effect [10] (which can be obtained from those above by truncating the series to $k = 2$, that is taking $\mathcal{N}^{[p]}_{2}(\xi) = 1$). Similar factors also appear in optomechanics [11].

With the above notations we obtain

$$\tilde{a}[\xi] = -\frac{\sqrt{\kappa}\chi(\xi)}{1 - \sum_{p=1}^{2}\mathcal{N}^{[p]}_{1}(\xi)}\left\{\tilde{a}_{\rm in}[\xi] + \sum_{p=1}^{2}\frac{\alpha_p\chi^*(2\Delta_p - \xi)}{\mathcal{N}^{[p]}_{2}(\xi)}\left\{(\tilde{a}_{\rm in}[2\Delta_p - \xi])^\dagger + \frac{\alpha^*_{\bar{p}}\chi(2\Delta_{\bar{p}} - 2\Delta_p + \xi)}{\mathcal{N}^{[p]}_{3}(\xi)}\times \right.\right.$$
$$\left.\left.\times\left\{\tilde{a}_{\rm in}[2\Delta_{\bar{p}} - 2\Delta_p + \xi] + \frac{\alpha_p\chi(4\Delta_p - 2\Delta_{\bar{p}} - \xi)}{\mathcal{N}^{[p]}_{4}(\xi)}\left\{(\tilde{a}_{\rm in}[4\Delta_p - 2\Delta_{\bar{p}} - \xi])^\dagger + \dots\right\}\right\}\right\}\right\}. \tag{12}$$

Essentially, Eq. (12) is the solution of our problem: it allows us to calculate the field inside the cavity at a frequency ξ from knowing the input field at all other frequencies. The formula Eq. (12) is clearly a generalization of the DCE formula for a single pump, see Eq. (4) from Ref. 10, enabling us to calculate the measured output field by using the connection formula

$$\tilde{a}_{\rm out}[\xi] = \tilde{a}_{\rm in}[\xi] + \sqrt{\kappa}\tilde{a}[\xi]. \tag{13}$$

The expansion behaves numerically very well, providing convergent results when used to calculate for example correlations and power levels. This is the case if the pumping powers are below the stability threshold values, defined by the condition that the absolute value of all the denominators is not zero. In particular, we have tested numerically the unitarity of this transformation, Eq. (12), $\left[\tilde{a}_{\rm out}[\xi], (\tilde{a}_{\rm out}[\xi'])^\dagger\right] = \delta(\xi - \xi')$, provided that the input fields satisfy the standard commutation relations. Also, one notices that for large values of ξ, $\xi \gg \Delta_1, \Delta_2, \kappa$, we have $\tilde{a}^{\rm (E)}_{\rm out}[\xi] \approx \tilde{a}^{\rm (E)}_{\rm in}[\xi]$, as expected. Finally, ignoring irrelevant phase factors, the basic structure of Eq. (12) is $a_{\rm out}[\xi] = g(\xi)a_{\rm in}[\xi] + \mathcal{F}[\xi]$, with the added-noise operator $\mathcal{F}$ collecting all the contributions at $2\Delta_p - \xi$, $2\Delta_{\bar{p}} - 2\Delta_p + \xi$, $4\Delta_p - 2\Delta_{\bar{p}} - \xi$, *etc.*. This can be seen as a phase-insensitive (phase-preserving) amplifier with gain

$$G(\xi) = |g(\xi)|^2 = \left|1 - \frac{\kappa\chi(\xi)}{1 - \sum_{p=1}^{2}\mathcal{N}^{[p]}_{1}(\xi)}\right|^2. \tag{14}$$

Dissipation can be modeled by introducing a fictive internal port I, distinct from the external port E used for out-coupling the field into the measurement chain. With the internal part, the total

decay rate κ consists of an internal component $\kappa_{\rm I}$ and an external component $\kappa_{\rm E}$, with $\kappa = \kappa_{\rm E} + \kappa_{\rm I}$, and the total input and output fields at any frequency is given by

$$\tilde{a}_{\rm in}[\xi] = \sqrt{\frac{\kappa_{\rm E}}{\kappa}}\tilde{a}_{\rm in}^{(\rm E)}[\xi] + \sqrt{\frac{\kappa_{\rm I}}{\kappa}}\tilde{a}_{\rm in}^{(\rm I)}[\xi], \tag{15}$$

$$\tilde{a}_{\rm out}[\xi] = \sqrt{\frac{\kappa_{\rm E}}{\kappa}}\tilde{a}_{\rm out}^{(\rm E)}[\xi] + \sqrt{\frac{\kappa_{\rm I}}{\kappa}}\tilde{a}_{\rm out}^{(\rm I)}[\xi]. \tag{16}$$

Note that this is a unitary transformation of the beam-splitter type, with probabilities $\kappa_{\rm E}/\kappa$ and $\kappa_{\rm I}/\kappa$ for a photon to enter/exit the cavity. Knowing $a(t)$ we can obtain the output field in the external mode,

$$\tilde{a}_{\rm out}^{(\rm E)}[\xi] = \tilde{a}_{\rm in}^{(\rm E)}[\xi] + \sqrt{\kappa_{\rm E}}\tilde{a}[\xi], \tag{17}$$

and similarly for the internal mode. When referenced to the external mode, the gain becomes

$$G^{(\rm E)}(\xi) = \left|1 + \frac{\kappa_{\rm E}\chi(\xi)}{1 - \sum_{p=1}^{2}\mathcal{N}_1^{[p]}(\xi)}\right|^2. \tag{18}$$

Supplementary Note 3: Correlations

To understand the origin and significance of the correlations in this problem, it is better to work with the first-order reflections rather than the full result Eq. (12). Let us truncate the series Eq. (12) to the first three terms only. This corresponds also to the real experimental situation, where only these terms are seen clearly at low pumping powers. One can see also from Fig. Supplementary Figure 1 that, especially if ξ is in-between the pumps $\Delta_1 < \xi < \Delta_2$ and not too large (that is, close to the cavity resonance), then the second-order reflections at $2\Delta_1 - 2\Delta_2 + \xi$ and $2\Delta_2 - 2\Delta_1 + \xi$ are rather far away. According to Eq. (12), their input fields will be multiplied by the cavity susceptibility at these frequencies - thus they will be much smaller than the first-order reflections.

We parametrize the pump amplitudes α_1 and α_2 using

$$\alpha_1\chi^*(2\Delta_1) = A\cos\theta e^{i\varphi_1}, \tag{19}$$

$$\alpha_2\chi^*(2\Delta_2) = A\sin\theta e^{i\varphi_2}. \tag{20}$$

Here the asymmetry between the pump strengths is accounted for by the angle θ and A is a positive real number characterizing the overall pumping amplitude. For convenience, we took here the variable ξ as referring to frequencies near the origin, $|\xi| \ll |\Delta_p|$, which yields $\chi^*(2\Delta_p - \xi) \approx \chi^*(2\Delta_p)$. Next, we introduce two modes, a 'bright' one $\tilde{b}$ and a 'dark' one $\tilde{d}$, defined by

$$\tilde{b}[\xi] = e^{-i\varphi_1} \cos\theta \tilde{a}[2\Delta_1 - \xi] + e^{-i\varphi_2} \sin\theta \tilde{a}[2\Delta_2 - \xi]; \tag{21}$$

$$\tilde{d}[\xi] = e^{-i\varphi_1} \sin\theta \tilde{a}[2\Delta_1 - \xi] - e^{-i\varphi_2} \cos\theta \tilde{a}[2\Delta_2 - \xi]. \tag{22}$$

These two modes are orthogonal to each other. Note that, the definition of modes $\tilde{b}$ and $\tilde{d}$ resembles a beam-splitter operation in frequency space, where a mode is separated into two branches with a distance $2(\Delta_1 - \Delta_2)$ between them.

With these notations we can calculate $\tilde{a}_{\text{out}}[\xi]$ using Eq. (12). In order to make the mathematical structure even more transparent, we can further simplify the result by neglecting the terms $\xi/\kappa \ll 1$ and $(\Delta_1 \cos^2\theta + \Delta_2 \sin^2\theta)/\kappa$ that appear in the normalization factor. The justification of this approximation is that, in order to be able to measure any signal at all, the detunings Δ_1, Δ_2, and ξ have to be in general within the decay rate of the cavity, otherwise they will be filtered out. With this approximation ($\xi \ll \Delta_p \ll \kappa$) we get

$$\tilde{a}_{\text{out}}[\xi] \simeq -\frac{1 + A^2}{1 - A^2} \tilde{a}_{\text{in}}[\xi] - \frac{2A}{1 - A^2} \left(\tilde{b}_{\text{in}}[\xi]\right)^\dagger. \tag{23}$$

Eq. (23) allows us to identify the stability condition $A(\xi) < 1$. The structure of Eq. (23) suggests the parametrization $A = \tanh(\lambda/2)$, which results in

$$\tilde{a}_{\text{out}}[\xi] \simeq -\cosh\lambda \tilde{a}_{\text{in}}[\xi] - \sinh\lambda \left(\tilde{b}_{\text{in}}[\xi]\right)^\dagger. \tag{24}$$

Similarly, to find the output $\tilde{b}$ mode, we write the result Eq. (12) at $2\Delta_1 - \xi$ and $2\Delta_2 - \xi$, neglect the fields at $2\Delta_1 - 2\Delta_2 + \xi$, $2\Delta_2 - 2\Delta_1 + \xi$ and beyond, and use the definition Eq. (21). Within the same approximation as above, we obtain

$$\tilde{b}_{\text{out}}[\xi] \simeq -\cosh\lambda \tilde{b}_{\text{in}}[\xi] - \sinh\lambda \left(\tilde{a}_{\text{in}}[\xi]\right)^\dagger. \tag{25}$$

Let us assume now zero temperature limit, a condition which is satisfied in our experiment. Then we obtain

$$\left\langle \tilde{a}_{\rm out}[\xi]\tilde{a}_{\rm out}[2\Delta_1-\xi']\right\rangle \;=\; \frac{e^{i\varphi_1}}{2}\sinh 2\lambda\cos\theta\times\delta(\xi-\xi'), \tag{26}$$

$$\left\langle \tilde{a}_{\rm out}[\xi]\tilde{a}_{\rm out}[2\Delta_2-\xi']\right\rangle \;=\; \frac{e^{i\varphi_2}}{2}\sinh 2\lambda\sin\theta\times\delta(\xi-\xi'), \tag{27}$$

$$\left\langle (\tilde{a}_{\rm out}[2\Delta_1-\xi])^\dagger\tilde{a}_{\rm out}[2\Delta_1-\xi']\right\rangle \;=\; \sinh^2\lambda\cos^2\theta\times\delta(\xi-\xi'), \tag{28}$$

$$\left\langle (\tilde{a}_{\rm out}[2\Delta_2-\xi])^\dagger\tilde{a}_{\rm out}[2\Delta_2-\xi']\right\rangle \;=\; \sinh^2\lambda\sin^2\theta\times\delta(\xi-\xi'), \tag{29}$$

$$\left\langle (\tilde{a}_{\rm out}[\xi])^\dagger\tilde{a}_{\rm out}[\xi']\right\rangle \;=\; \sinh^2\lambda\times\delta(\xi-\xi'), \tag{30}$$

and in terms of the bright mode,

$$\left\langle \tilde{a}_{\rm out}[\xi]\tilde{b}_{\rm out}[\xi']\right\rangle \;=\; \frac{1}{2}\sinh 2\lambda\times\delta(\xi-\xi'), \tag{31}$$

$$\left\langle (\tilde{b}_{\rm out}[\xi])^\dagger\tilde{b}_{\rm out}[\xi']\right\rangle \;=\; \sinh^2\lambda\times\delta(\xi-\xi'). \tag{32}$$

By contrast, applying the same procedure to the dark mode results in

$$\left\langle \tilde{a}_{\rm out}[\xi]\tilde{d}_{\rm out}[\xi']\right\rangle \;=\; \left\langle \tilde{b}_{\rm out}[\xi]\tilde{d}_{\rm out}[\xi']\right\rangle = 0, \tag{33}$$

$$\left\langle (\tilde{d}_{\rm out}[\xi])^\dagger\tilde{d}_{\rm out}[\xi']\right\rangle \;=\; 0. \tag{34}$$

The dissapearance of noise power in the dark mode is clearly a coherence effect, which is conceptually different from destructive interference. Indeed, the two modes at $2\Delta_1-\xi$ and $2\Delta_2-\xi$ are separated in frequency and they do not really come together to overlap. The coherence is created by the vacuum fluctuations at ξ which triggers correlated spontaneous parametric downconversion two-photon processes in the pumps.

As in the usual interference phenomena, there exist also coherent terms that depend on the phase of the pumps,

$$\left\langle (\tilde{a}_{\rm out}[2\Delta_2-\xi])^\dagger\tilde{a}_{\rm out}[2\Delta_1-\xi']\right\rangle \;=\; \frac{1}{2}e^{i(\varphi_1-\varphi_2)}\sin 2\theta\sinh^2\lambda\times\delta(\xi-\xi'), \tag{35}$$

$$\left\langle (\tilde{a}_{\rm out}[2\Delta_1-\xi])^\dagger\tilde{a}_{\rm out}[2\Delta_2-\xi']\right\rangle \;=\; \frac{1}{2}e^{-i(\varphi_1-\varphi_2)}\sin 2\theta\sinh^2\lambda\times\delta(\xi-\xi'). \tag{36}$$

All these correlations can be extracted from the experimental data by using the field quadratures for the output field, defined at each frequency, ξ, $2\Delta_1-\xi$, and $2\Delta_2-\xi$,

$$x_{\rm out} \;=\; \frac{1}{2}\left(a_{\rm out}+a_{\rm out}^\dagger\right), \tag{37}$$

$$y_{\rm out} \;=\; \frac{1}{2i}\left(a_{\rm out}-a_{\rm out}^\dagger\right), \tag{38}$$

which, when convoluted with the filtering function, give the I-Q quadratures measured in the experiment, see Methods.

Supplementary Note 4: Tripartite structure for the parametric vacuum-induced coherence effect

To have a physical understanding of the origin of these correlations, we use the powerful principle of which-way information introduced in quantum optics and used to interpret many fundamental experiments in optics [2]. Our work provides a fully new system to apply these concepts, namely a microwave setting at very low temperatures. As always in microwave-based measurements, we cannot do single-photon experiments due to the lack of single-photon detectors; also we do not have the possibility to change the detection settings on the fly. Still, our setup brings in a fundamentally new feature: the number of modes of interest is only three (with the approximations used above; small amplitude and $\xi \ll \Delta_p$), while in the corresponding quantum optics experiments it is usually four (two nonlinear crystals with one idler and one signal each). For example, in the experiments on entanglement swapping [3], one mode from each of the nonlinear crystals were combined and a Bell-state projection on these two modes was performed; the photons in the other two modes became entangled. Spectacularly, the choice of Bell state projection could be made after the photons had been "born" through the corresponding down-conversion processes. Even closer to our experiment, Ref. [4] used a single crystal to produce the photons. Although, even in this case there were four modes, two of which were combined in a beam splitter.

Suppose that we would have a detector with a frequency filter, capable of detecting photons only in a relatively narrow frequency interval. This leads naturally to the concept of frequency-bin entanglement (color entanglement). For clarity, let us restrict the discussion to the subspace of single photons: in other words, we assume that the pumps have so low intensity that only one downconversion process occurs at a time, resulting in two photons in the resonator. Quantum-optics experiments having similarity with our correlation experiments have been performed using an interferometer developed by Mandel [5]. In a series of experiments in the early 1990's, Mandel showed that by aligning the idler beams from two nonlinear crystals, where spontaneous parametric downconversion occurs, it is possible to induce coherence between the two remaining signal beams. More recently, Zeilinger's group in Vienna demonstrated that this principle can be used for optical imaging with undetected photons [6]. In these experiments, it is the lack of which-path information that created the interference in the signal beams. In our case, there is no path in real space. Instead, we can talk about **which-color information**: for a real photon, at frequency ξ, there is no way to

know from which of the two spontaneous parametric downconversion process around Δ_1 or Δ_2 it came from. Another peculiarity of our system is that there are not only two downconversions, but an infinite sequence of them: the mode ξ acts as the common idler for both pumps, producing two signals, which in turn act as the idlers for the pumps where they did not originate, *etc.*.

To discuss the connection with these fundamental aspects of few-photons physics, let us consider only the first-order reflections and simplify the notations by using the bandwidth- averaged modes $\tilde{a}_0$, $\tilde{a}_1$, and $\tilde{a}_2$. Suppose we work at such low intensities that at most two photons of either of the frequencies $\xi, 2\Delta_1 - \xi, 2\Delta_2 - \xi$ are present in the system at a time. One of these photons must always be in the mode $\tilde{a}_0$: otherwise, the two photons should be one in $\tilde{a}_1$, the other in $\tilde{a}_2$, and there is no term in the Hamiltonian that corresponds to this process. Now, for the photon at $\tilde{a}_0$ there is no way, even in principle, of determining where it came from - pump 1 or pump 2. The probabilities of originating from the downconversions in either one of these pumps might be different (for example if the pumps have different intensities) but for a particular occurrence of a photon in $\tilde{a}_0$ there is nothing distinctive to allow us to say where it came from. This lack of which-way information implies that, in the two-photon subspace, the state of the resonator at each frequency ξ must be a superposition,

$$|\psi\rangle \;=\; \frac{1}{\sqrt{2}}\left(|1\rangle_{\tilde{a}_1}|1\rangle_{\tilde{a}_0}|0\rangle_{\tilde{a}_2} + |0\rangle_{\tilde{a}_1}|1\rangle_{\tilde{a}_0}|1\rangle_{\tilde{a}_2}\right) = |\beta\rangle_{\tilde{a}_1;\tilde{a}_2}|1\rangle_{\tilde{a}_0}\,, \tag{39}$$

where $|\beta\rangle_{\tilde{a}_1;\tilde{a}_2} = (1/\sqrt{2})\left(|1\rangle_{\tilde{a}_1}|0\rangle_{\tilde{a}_2} + |0\rangle_{\tilde{a}_1}|1\rangle_{\tilde{a}_2}\right)$ is a Bell state. For simplicity, we assume here that the two processes have equal probability amplitude, which can be achieved by considering two pumps with properly adjusted amplitude and phase. Thus, even in the low-intensity limit, one does expect the appearance of correlations between the photons in the modes $\tilde{a}_1$ and $\tilde{a}_2$.

The appearance of the state Eq. (39) can be obtained more formally by analyzing the structure of the input-output equations. Let us consider a resonator with a large enough external coupling κ, ensuring that the field is nonzero at some finite detuning, in other words we take $|\xi|, \Delta_1, \Delta_2 \ll \kappa$. Then, we can write Eqs. (24,25) as

$$\tilde{a}_{0,\mathrm{out}} \;\approx\; -S^{\dagger}\tilde{a}_{0,\mathrm{in}}S, \tag{40}$$

$$\tilde{b}_{\mathrm{out}} \;\approx\; -S^{\dagger}\tilde{b}_{\mathrm{in}}S, \tag{41}$$

where the squeezing transformation S is defined as

$$S = \exp\left[\lambda\tilde{a}_{0,\mathrm{in}}\tilde{b}_{\mathrm{in}} - h.c.\right]. \tag{42}$$

This imposes a quite specific structure for the output vacuum

$$|0\rangle_{\rm out} = S^{\dagger}\,|0\rangle_{\rm in}\,, \tag{43}$$

which follows from the requirements $\tilde{a}_{j,\rm in}\,|0\rangle_{\rm in} = 0$ $\tilde{a}_{j,\rm out}\,|0\rangle_{\rm out} = 0$, where $j = 0, 1, 2$. When the pumps have identical amplitudes and phases, from Eq. (43) it follows immediately that the output state in the subspace with two photons has the structure Eq. (39), which was derived from general quantum-information concepts. The state Eq. (43) is a two-mode squeezed state when considering the modes $(\tilde{a}_{1,\rm in} + \tilde{a}_{2,\rm in})/\sqrt{2}$ and $\tilde{a}_{0,\rm in}$, while in the subspace of the modes $\tilde{a}_{1,\rm in}$ and $\tilde{a}_{2,\rm in}$ the double pumping yields a beam-splitter transformation, producing a superposition. This is called a bisymmetric state[7, 8]. When truncated to the subspace containing no more than two photons (a regime that can be achieved experimentally by lowering the pump powers), the state obtained from Eq. (43) is a superposition of the vacuum and the two-photon state Eq. (39). This is a tripartite state in the W class [9], as can be checked by applying an X gate to the photon in mode $\tilde{a}_0$.

Supplementary Note 5: Single-photon effects

The coherence effect seen in the experiment has a quantum-mechanical origin. It stems from the wavelike-character of quantum particles, which cannot be seen here as discrete lumps of energy that are randomly created by parametric downconversion processes occuring in each pump separately. To see this more clearly, we will rule out one by one all reasonable semiclassical type of explanation based on the particle character of the photons. First, let us introduce the cavity density of states

$$\mathcal{D}(\xi) = \frac{\kappa}{2\pi}|\chi(\xi)|^2. \tag{44}$$

As expected, in a lossless cavity $\mathcal{D}(\xi) = \delta(\xi)$, corresponding to a single mode at the resonance frequency. With this notation, we now investigate the power spectrum produced by a single parametric downconversion process, which can be obtained immediately from our general result Eq. (12) by switching off one of the pumps,

$$\mathrm{DCE}_p(\xi) = 4\pi^2|\alpha_p|^2\mathcal{D}(\xi)\mathcal{D}(2\Delta_p - \xi), \tag{45}$$

with $p = 1, 2$. This of course is the result for the single-pump dynamical Casimir effect (DCE), see Eq. (1) in Ref. 10. The quantity $\mathrm{DCE}_p(\xi)$ is the adimensional noise spectrum that specifies how much power $< \tilde{a}^{\dagger}_{\rm out}\tilde{a}_{\rm out} >$ is in the output mode relative to the background vacuum level, at

frequency ξ. Here $|\alpha_p|$ is the rate of pump downconversion from the Hamiltonian Eq. (3). The rate of power conversion from the pump (the rate of pump depletion) is then $|\alpha_p|^2$, which has to be multiplied with the densities of states at the frequency of the idler and of the signal.

The coherence persists down to average cavity photon numbers of the order of unit and even below, see Eq. (39). This equation shows that the superposition in the bright mode exists even for a single photon present in the modes $\tilde{a}_{1,\mathrm{in}}$ and $\tilde{a}_{2,\mathrm{in}}$. This is important to understand, because one might try the following semi-classical description: suppose that one spontaneous parametric downconversion process occurs in pump 1: as a result, two photons are created, one at ξ and the other at $2\Delta_1 - \xi$. Next, the photon at ξ acts as signal for the second pump, which triggers another downconversion process, with the creation of another pair, with one photon at ξ and another one at $2\Delta_2 - \xi$. Thus, the minimum number of photons required would be four, and also this process would be second-order: its probability would depend on the product of the squared pump amplitudes. This would imply that the total power spectrum is $\mathrm{DCE}_1(\xi) \times \mathrm{DCE}_2(\xi) \approx |\alpha_1|^2|\alpha_2|^2$. This is clearly not the case, and the formalism developed in Sect. III shows that the process depends linearly on the power of the pumps.

Our doubly pumped DCE system illustrates some of the standard paradoxes of quantum-mechanical interference in a new setting. Let us postulate that we would be in possession of a single-photon detector, with enough frequency resolution so that it could discriminate between the frequencies ξ, $2\Delta_1 - \xi$, and $2\Delta_2 - \xi$. Such a detector, when placed at the output of the cavity, would detect one by one the photons, as they were produced in the cavity. This would lead immediately to an apparent paradox: as the detector would detect a photon at the frequency $2\Delta_1 - \xi$, we could infer that this was the result of a parametric downconversion in pump 1, accompanied by the creation of a photon at ξ. This would ensure energy conservation: a downconversion in pump 2 would have produced one photon at energy ξ and another one at $2\Delta_2 - \xi$, and the sum of these frequencies would equal $2\Delta_2$ instead of the required $2\Delta_1$. This means that we were able to extract **which-color information**, and as a result the coherence, when counting large enough number of photons, should disappear. The situation is analogous to the vanishing of the interference fringes in a two-slit interferometer where the transversal momentum kick onto the panel with the slits is measured in order to extract which-way information. According to this reasoning, the interference should not exist even in the case of our experiment, simply because it would be in principle possible to use a single-photon detector.

The apparent paradox discussed above is solved immediately when we apply the time-energy uncertainty principle to the resonator. The decay time of the cavity sets the time uncertainty to $\delta t \sim \kappa^{-1}$, which yields $\Delta E \gtrsim \hbar\kappa$. This means that it is not possible to infer from the existence of a detection event at $2\Delta_1 - \xi$ that the downconversion occurred in pump 1. Attempting to increase the quality factor of the cavity (while keeping the detunings Δ_1 and Δ_2 fixed) will result in the overall loss of signal at ξ, $2\Delta_1 - \xi$, and $2\Delta_2 - \xi$. Thus the coherence does eventually dissapear while attempting to extract which-color information, though the mechanism is somewhat different from the case of standard optical interferometer experiments.

Supplementary Note 6: Pulsed parametric pumping

We analyze here the case when the pumps act only during finite time intervals T_p, $p = 1, 2$ centered at delay times τ_p, respectively. In other words, the pumping amplitudes α_p can be approximated as rectangular pulses

$$\alpha_p(t) = \begin{cases} |\alpha_p| e^{i\phi_p}, & t \in \left[\tau_p - \frac{T_p}{2}, \tau_p + \frac{T_p}{2}\right] \\ 0, & \text{otherwise} \end{cases} \tag{46}$$

The Fourier transform is

$$\alpha_p[\xi] = \frac{1}{\sqrt{2\pi}} \int_{-\infty}^{\infty} dt e^{i\xi t} \alpha(t) = \frac{|\alpha_p| e^{i(\xi\tau_p + \phi_p)}}{\sqrt{2\pi}} T_p \,\mathrm{sinc}\left(\frac{\xi T_p}{2}\right), \tag{47}$$

where the sinc function is defined as $\mathrm{sinc}(x) = \sin(x)/x$. We now wish to solve approximately the Heisenberg-Langevin equation with time-dependent pumps,

$$\dot{\tilde{a}} = \sum_{p=1,2} \alpha_p(t) e^{-2i\Delta_p t} \tilde{a}^\dagger - \frac{\kappa}{2}\tilde{a} - \sqrt{\kappa} a_{in}. \tag{48}$$

Taking the Fourier transform we obtain

$$\begin{aligned} &\left(\frac{\kappa}{2} - i\xi\right) \tilde{a}[\xi] - \frac{1}{\sqrt{2\pi}} \sum_{p=1,2} \int_{-\infty}^{\infty} d\xi'_p \alpha_p[\xi'_p] \tilde{a}^\dagger \left[\xi - 2\Delta_p - \xi'_p\right] \\ &= -\sqrt{\kappa}\tilde{a}_{\mathrm{in}}[\xi], \end{aligned} \tag{49}$$

where the effect of parametric pumping on the modes is obtained as a convolution. Next, we write this equation at the first reflections $2\Delta_p - \xi$ with respect to the pumps,

$$\begin{aligned}
&\left[\frac{\kappa}{2} - i(2\Delta_p - \xi)\right] \tilde{a}[2\Delta_p - \xi] \\
&-\frac{1}{\sqrt{2\pi}} \int_{-\infty}^{\infty} d\xi_p'' \alpha_p[\xi_p''] \tilde{a}^\dagger \left[-\xi - \xi_p''\right] \\
&-\frac{1}{\sqrt{2\pi}} \int_{-\infty}^{\infty} d\xi_{\bar{p}}'' \alpha_{\bar{p}}[\xi_{\bar{p}}''] \tilde{a}^\dagger \left[2(\Delta_p - \Delta_{\bar{p}}) - \xi - \xi_{\bar{p}}''\right] \\
&= -\sqrt{\kappa}\tilde{a}_{\text{in}}[\xi]. \qquad (50)
\end{aligned}$$

We can extract $\tilde{a}[2\Delta_p - \xi]$, $p = 1, 2$ from Eq. (50) (we neglect the second-order reflections with respect to the pumps) and we insert them to Eq. (49). This results in an integral equation for $a[\xi]$, which can be solved approximatively under the assumption that the second-order terms in the pump amplitudes are small:

$$\left|\frac{\kappa}{2} - i\xi\right| \gg \int_{\infty}^{\infty} d\xi_p' \int_{-\infty}^{\infty} d\xi_p' \left|\frac{\alpha_p[\xi_p']\alpha_p^*[\xi_p'']}{\frac{\kappa}{2} + i(2\Delta_p - \xi + \xi_p')}\right|.$$

This corresponds to the regime of small gain, similar to a linear approximation in λ in the continuous case discussed before $\cosh(\lambda) \approx 1$, $\sinh(\lambda) \approx \lambda$. Using the cavity susceptibility $\chi(\xi) = (\kappa/2 - i\xi)^{-1}$, we can estimate

$$\begin{aligned}
&\tilde{a}_{\text{out}}[\xi] \approx -[-1 + \kappa\chi(\xi)]\tilde{a}_{\text{in}}[\xi] \\
&-\frac{\kappa\chi(\xi)}{\sqrt{2\pi}} \sum_{p=1,2} \int_{-\infty}^{\infty} d\xi_p' \alpha_p[\xi_p'] \chi^*(2\Delta_p - \xi + \xi_2') \times \\
&\times \tilde{a}_{\text{in}}^\dagger[\xi - \xi_p' - 2\Delta_p]. \qquad (51)
\end{aligned}$$

One can notice that, indeed, the first term after the approximately sign yields a gain of 1 and a phase shift which corresponds to the well-known reflection $-(\kappa/2 + i\xi)/(\kappa/2 - i\xi)$ from a cavity in the absence of pumping. Using this expression, we can calculate the vacuum-induced correlation

$$\langle (a_{\text{out}}[2\Delta_1 - \xi_1])^\dagger a_{\text{out}}[2\Delta_2 - \xi_2] \rangle \approx \frac{\kappa^2}{2} \chi^*(2\Delta_1 - \xi_1)\chi(2\Delta_2 - \xi_2) \int_{-\infty}^{\infty} d\xi_1'' \alpha_1^*(\xi_1'')\alpha_2(\xi_1'' + \xi_1 - \xi_2)|\chi(\xi_1'' + \xi_1)|^2. \qquad (52)$$

We assume that the response of the cavity is rather flat $\chi \approx 2/\kappa$ in the region between $2\Delta_1$ and $2\Delta_2$, in other words $\kappa >> \Delta_p$. In this case $\phi_p \approx \varphi_p$ according to the definitions used before. We can use the fact that the convolution of two sinc functions is also a sinc function, and we find

$$\langle (a_{\text{out}}[2\Delta_1 - \xi_1])^\dagger a_{\text{out}}[2\Delta_2 - \xi_2] \rangle \approx \frac{16\kappa^2}{2\pi} |\alpha_1||\alpha_2| T_0 \,\text{sinc}\left[\frac{(\xi_1 - \xi_2)T_0}{2}\right] e^{i(\xi_1 - \xi_2)\tau_0 + \varphi_2 - \varphi_1}, \qquad (53)$$

where T_0 is the duration of overlap of the two pulses and τ_0 is the center of this overlap. Assuming $\tau_2 > \tau_1$,

$$T_0 = \frac{\tau_1 - \tau_2}{2} + \frac{T_1 + T_2}{2}, \tag{54}$$

$$\tau_0 = \frac{\tau_1 + \tau_2}{2} + \frac{T_1 - T_2}{4}. \tag{55}$$

In the experiment, this correlation is calculated at $\xi_1 \approx \xi_2$ where it reaches the maximal value $16\kappa^{-2}A_1A_2T_0/2\pi$. This implies a linear decrease of this correlation as a function of the overlap T_0, which, if τ_1 is fixed, decreases linearly in the offset τ_2 of the second pulse from its maximum value to zero, when the pulses do not overlap. This dependence is precisely what is observed in the experiment (see Fig. 5 of the main paper).

Note that in the case of large T_0 we recover the results of continuous pumping, by using the sinc representation of the Dirac function,

$$\delta_\epsilon(x) = \frac{\sin(x/\epsilon)}{\pi x}, \tag{56}$$

with $\epsilon \to 0$. When applied to Eq. (52) we get the previous result,

$$\langle (a_{\text{out}}[2\Delta_1 - \xi_1])^\dagger a_{\text{out}}[2\Delta_2 - \xi_2] \rangle \approx \frac{16}{k^2}|\alpha_1||\alpha_2|e^{i(\varphi_2-\varphi_1)}\delta(\xi_1 - \xi_2). \tag{57}$$

Supplementary Note 7: Additional information about the experiment and the sample

Our LC-resonator, formed by the capacitively shunted SQUID, is coupled to the 50 Ω environment of our measurement system. A natural impedance mismatch exists due to the resonator impedance which is on the order of $\sqrt{L/C} \approx 1$ Ω. Overall, this results in a weak coupling between the resonator dissipation and the measurement system. Additionally the bonding wires also provide a series inductance of approximately 2 nH which influences the device. The SQUID has a nominal critical current of 66 μA. In the experiment this value is tuned down from the maximum value (beyond our measurement range) to 5 GHz by applying magnetic flux.

To guarantee the thermalization of noise entering the sample, all lines down to the experimental space were strongly attenuated, with attenuation $\alpha > 100$ dB in the flux-bias dc-feed line, $\alpha > 70$ dB in the microwave cables for probe signals, and $\alpha = 40$ dB in the pump line. A high pass filter with $f_c = 8$ GHz was installed in the pump line to prevent thermal noise at signal frequencies from

reaching the sample. Off-band noise from the LNA input was isolated by a bandpass filter operating in the 4-8 GHz frequency range. Two circulators at the mixing chamber plate were used: they provide an isolation on the order of 40 dB from the LNA input at 4 K. In Supplementary Figure 2 we present the results of the reflection measurement used to extract the external and internal cavity rates introduced in Eqs. 15-17.

A schematic of the essential parts of the measurement setup is depicted in Fig. Supplementary Figure 3, while the sample details are illustrated in Supplementary Figure 4. Supplementary Figure 5 and Supplementary Figure 6 provide additional results on the correlators and the measured noise at large pumping.

The experiment relied on three phase-coherent sources, the two pumps from two low phase noise microwave generators and the LO of the signal analyzer. The coherence provided by the common 10 MHz frequency reference from a rubidium standard provided sufficient stability for obtaining the results presented in this paper.

Supplementary Note 8: Additional information about data analysis

The signal analyzer digitizes $I(t)$ and $Q(t)$ at a sampling rate of 50 MHz which also yields a data bandwidth of 50 MHz (Nyquist limit for quadrature information). Additionally, the signal analyzer has a built-in sharp-edged hardware filter limiting the analog bandwidth to 31.25 MHz thus eliminating potential aliasing artifacts. Due to computing limitations (as well as hardware phase drift related issues) we transform and process at most $N = 2^{23} \approx 8$M samples at a time. Altogether we collect approximately 20 GB of data.

This data vector is Fourier transformed in the complex form yielding a complex vector $\mathcal{F}_1^N = \mathrm{FFT}(I + iQ)$ containing the amplitude and phase representation of the signal in frequency domain. Here subscript and superscript are used to indicate the index of the first and last element. This vector is constructed such that the first index (1) corresponds to the lowest frequency of 4975 MHz and the last index (N) corresponds to the highest frequency of 5025 MHz. The index corresponding to the frequency of interest can be linearly interpolated from this interval and the data from the desired bandwidth $k = b - a = d - c$ measured in indexes copied to new vectors $f_1^k = \mathcal{F}_a^b$ and $g_1^k = \mathcal{F}_c^d$, where $1 < a < b < N$. These vectors can be used to directly calculate the correlation in frequency space. Calculating the complex cross-correlation defined as $(f \star g)[n] = \sum_{m=1}^{k} f^*[m]g[m+n]$, yields in addition to magnitude, also the argument of the correlated signals respect to each other. The cross-correlation corresponding to two mode squeezing correlations is calculated as $(f^* \star \bar{g})[n]$, where $\bar{g}$ indicates a reversed vector such that $\bar{g}[m] = g[k - m + 1]$. Additionally, we can do an inverse FFT on these sliced data and transform the quadratures back into the time domain.

Two mode squeezing can be extracted by simply repeating the previously described procedure for two separate frequencies and calculating the correlation separately for all pairs and combinations of real and imaginary parts. This was done to obtain the histogram presented as the inset of Fig. 3. in the main paper.

Supplementary References

[1] Clerk, A. A., Devoret, M. H., Girvin, S. M., Marquardt, F. & Schoelkopf, R. J., *Rev. Mod. Phys.* **82**, 1155 (2010).

[2] Ma, X.-S., Kofler, J. & Zeilinger, A., arXiv:1407.2930.

[3] Ma, X.-S., Zotter, S., Koer, J., Ursin, R., Jennewein, T., Brukner, C. & Zeilinger, A., *Nature Phys.* **8**, 479 (2012).

[4] Kim, Y.-H., Yu, R., Kulik, S. P., Shih, Y. & Scully, M. O., *Phys. Rev. Lett.* **84**, 1 (2000).

[5] Zou, X. Y., Wang, L. J., Mandel, L., *Phys. Rev. Lett.* **67**, 318 (1991),

[6] Lemos, G. B., Borish, V., Cole, G. D., Ramelow, S., Lapkiewicz, R. & Zeilinger, A., *Nature* **512**, 409 (2014).

[7] Serafini, A., Adesso, G. & Illuminati, F., *Phys. Rev. A* **71**, 032349 (2005).

[8] Adesso, G., Serafini, A. & Illuminati, F. *Phys. Rev. Lett.* **93**, 220504 (2004).

[9] Dür, W., Vidal, G. & Cirac, J. I., *Phys. Rev. A* **62**, 062314 (2000).

[10] Lähteenmäki, P., Paraoanu, G. S., Hassel, J. & Hakonen, P. J., *Proc. Natl. Acad. Sci. U.S.A.* **110**, 4234 (2013).

[11] Marquardt, F., Chen, J. P., Clerk, A. A. & Girvin, S. M., *Phys. Rev. Lett.* **99**, 093902 (2007).
